# Indicators of the molecular pathogenesis of virulent Newcastle disease virus in chickens revealed by transcriptomic profiling of spleen

**DOI:** 10.1038/s41598-021-96929-w

**Published:** 2021-09-02

**Authors:** Mohammad Rabiei, Wai Yee Low, Yan Ren, Mohamad Indro Cahyono, Phuong Thi Kim Doan, Indi Dharmayanti, Eleonora Dal Grande, Farhid Hemmatzadeh

**Affiliations:** 1 School of Animal and Veterinary Sciences, The University of Adelaide, Adelaide, Australia; 2 The Davies Research Centre, School of Animal and Veterinary Sciences, The University of Adelaide, Adelaide, Australia; 3 Indonesian Research Centre for Veterinary Science, Bogor, West Java Indonesia; 4 Faculty of Animal and Veterinary Sciences, Tay Nguyen University, Daklak, Vietnam

**Keywords:** Immunology, Microbiology

## Abstract

Newcastle disease virus (NDV) has caused significant outbreaks in South-East Asia, particularly in Indonesia in recent years. Recently emerged genotype VII NDVs (NDV-GVII) have shifted their tropism from gastrointestinal/respiratory tropism to a lymphotropic virus, invading lymphoid organs including spleen and bursa of Fabricius to cause profound lymphoid depletion. In this study, we aimed to identify candidate genes and biological pathways that contribute to the disease caused by this velogenic NDV-GVII. A transcriptomic analysis based on RNA-Seq of spleen was performed in chickens challenged with NDV-GVII and a control group. In total, 6361 genes were differentially expressed that included 3506 up-regulated genes and 2855 down-regulated genes. Real-Time PCR of ten selected genes validated the RNA-Seq results as the correlation between them is 0.98. Functional and network analysis of Differentially Expressed Genes (DEGs) showed altered regulation of ElF2 signalling, mTOR signalling, proliferation of cells of the lymphoid system, signalling by Rho family GTPases and synaptogenesis signalling in spleen. We have also identified modified expression of *IFIT5*,* PI3K*,* AGT* and *PLP1* genes in NDV-GVII infected chickens. Our findings in activation of autophagy-mediated cell death, lymphotropic and synaptogenesis signalling pathways provide new insights into the molecular pathogenesis of this newly emerged NDV-GVII.

## Introduction

Newcastle disease virus (NDV) has a worldwide distribution. The NDV causes infection in many different avian species, and it can be considered a permanent threat to all poultry industries and other fields of aviculture^1^. NDV is capable of causing devastating infection for over 240 species of birds that can spillover through direct contact between healthy and infected birds^2,3^. NDV was first reported in Java, Indonesia in 1926 and then spread to the rest of the world. Various genotypes have been responsible for different ND panzootics^4^. Most recent ND outbreaks in Southeast Asia are mainly caused by highly virulent NDV-GVII leading to 70–80% mortality in commercial chickens, including vaccinated flocks^5,6^. While genotype VII NDVs includes a wide variety of sub-genotypes, the fourth and the fifth ND panzootic in Africa, Europe, Middle East and Asia were caused by genotype VII.1.1 (b, d, e, j, l) and VII.2 (a, h, i, k), respectively^6–8^.

NDV is a member of the genus avian *orthoavulavirus 1* within a new subfamily *Avulavirinae* of the family *Paramyxoviridae*^7^. NDV has a negative-sense single-stranded, and non-segmented RNA genome that encodes six major structural protein genes in the order 3′-NP-P-M-F-HN-L-5′^9^. According to the clinical signs in infected chickens, different strains of NDV have been separated into five groups^10^: (1) Viscerotropic velogenic strains causing acute lethal infections, usually causing haemorrhagic lesions in the intestines; (2) neurotropic velogenic strains causing high mortality with neurological disease followed by respiratory symptoms without gut lesions; (3) mesogenic strains causing low mortality with respiratory and neurological signs; (4) lentogenic strains causing mild infections of the respiratory tract without any lesions in the intestinal tract; and (5) avirulent strains that replicate in the intestine with no clinical signs. Avirulent strains are often used as live vaccines^6^. Phylogenetic analysis of the fusion protein gene of NDVs indicates that circulating strains in Indonesia are belonging to genotype VII.1 with a mean death time (MDT) from 33 and 30 h as their pathogenicity index^9,11,12^. These findings also indicate that these circulating strains are clinically categorised as velogenic. In this study, we have used one of these genotype VII NDV strains in our challenge experiment in order to analyse pathogenesis of this newly emerged NDV^11^. The amino acid composition of cleavage site of the fusion protein and its susceptibility to host trypsin-like proteases plays a big role in pathogenicity, spread of infection, and tissue tropism of NDVs^13^. Mucosal immunity is heavily involved in the host response to ND infection. Trachea, Harderian glands and lung are places for early virus/host contact at points of entry. These tissues are strategic sites to examine host–pathogen interaction and early viral shedding. Several studies investigated the transcriptome of these tissues infected by NDV^14–19^. In order to increase understanding of the immune response to NDV, the gene expression profile of other immune organs should also be considered. Recent in vivo studies revealed differential regulation of immune response to the lentogenic strain of NDV (LaSota) by transcriptome analysis in the spleen^20,21^. Another in vitro study compared transcript profile of highly virulent *Herts/33* strain and nonvirulent LaSota strain in spleen cells^22^. The transcriptomic analysis of infection caused by newly emerged virulent NDV-GVII has not been investigated in previous studies.

Cell death has been divided into three categories: (1) type I cell death or apoptosis; (2) type II cell death or necrosis; (3) type III cell death or autophagy^23^. Apoptosis is critical in both physiological and pathological conditions and is known as a multi-pathway process, which can lead to programmed cell death. Apoptosis is associated with many types of viral infections and, depending on the circumstances, can act to increase or decrease viral production. Apoptosis is a hallmark of cytotoxicity in virus-infected cells with NDV strains that can trigger both extrinsic and intrinsic apoptotic pathways^24^, and numerous in-vitro and in-vivo studies have shown that NDV can trigger the apoptosis process^25–28^. Different studies have shown that infection with the virulent strains of NDV will increase the apoptosis in lymphoid tissue and immune cells^25,29–31^. Severe splenic disruption, massive lymphoid depletion, ulceration of the intestinal epithelium and rapid depletion of the bursa of Fabricius have been described in association with these strains^25,29–32^. Other members of *Paramyxoviridae* family such as *Rinderpest*^33^, canine distemper, measles^34–36^, and *porcine Rubulavirus*^37^ similarly targeting the host lymphoid tissues. An important difference between apoptosis and necrosis is that apoptosis does not incite inflammation^28^.

The amino acid sequence motif ^112^RRQKRF^117^ of fusion protein has been previously indicated as the pathogenic genetic markers of virulent NDV^5,38,39^. Our previous studies have discovered and reported two different sub-genotypes of NDV GVII that contain RRQKRF motif in Mega strain and RRRKRF in Cimanglid and VD strains^9,11,12^. The Mega strain used in our challenge experiment carries the RRQKRF motif sequence in the fusion protein, and has been reported as pathonenicity indicator of the NDV even in genetically modified lentogenic strains^40,41^. In addition to these references, the Mega strain has been isolated from a brain of a dead chicken^11^. Based on all of the evidences, we were expecting to observe neurological lesions and respiratory symptoms as the classical symptoms of virulent NDV strains. Surprisingly, the virus load at the central nervous system of experimentally infected chickens was low or even zero in qPCR tests, while massive lymphoid depletion and high virus load were observed in studied chickens.. In this study, we aimed to identify the molecular basis of pathogenesis of newly emerged NDV-GVII using mRNA profiling of spleen tissues in experimentally infected chickens. To do that, we have focused on cell death related pathways and functional analysis of genes to reveal their potential roles in massive cellular depletion in spleen lymphoid tissues. To our knowledge, this is the first in vivo study investigating gene expression profile of this velogenic strain.

## Results

### Detection of virus shed in challenged group

To examine effects of virus on experimentally challenged birds, the Ross broiler Specific Pathogen Free (SPF) chickens were inoculated with a genotype VII NDV. Due to the severe sickness caused by the virus in challenged group, experiment terminated by euthanising of all birds at 2 dpi and a cloacal swab has also been taken from all birds including control group. The results of absolute quantification for detection of viral fusion gene in samples by qPCR confirmed that all the birds inoculated with NDV-GVII became infected and shed virus at 2 dpi. The mean cycle threshold (*Ct*) value of the challenge group (16.9, SD = 1.22) was lower than the control group (41.9 SD = 2.92) (F = 967.4, df = 14, p < 0.001), which indicated viral shedding in the challenged group while there was no detection of NDV in control group.

### Gene expression changes induced by NDV infection

**Figure 1:**
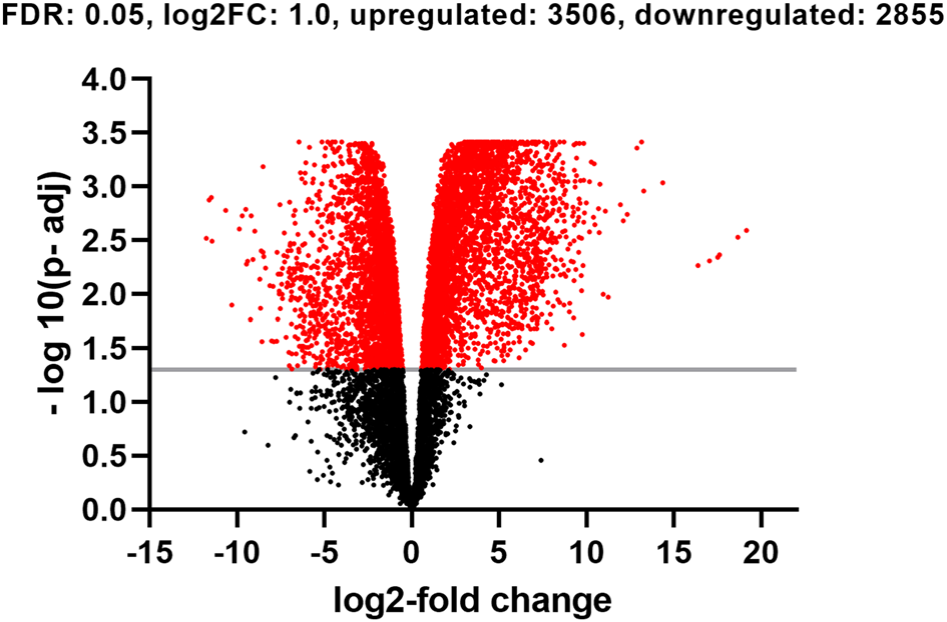
The volcano plot of differentially expressed genes between challenged and control birds. Red dots indicate significantly up-regulated (p < 0.05, log2 fold change ≥ 1) and down-regulated genes (p < 0.05, log_2_ fold change ≤  − 1). Black dots represent genes that were not differentially expressed.

Sequencing of constructed libraries from RNA samples resulted 400 million of 100-bp paired end reads. Similar percentages of reads from each sample (on average 76%) were mapped to the GRCg6a reference genome in the Ensembl database and could be counted as a gene feature by the software Feature Count (Table 1). Of the 24,362 annotated genes in the reference genome, 14,664 (~ 60%) genes were considered as expressed after our count per million cut-off criteria. By applying the FDR p-value cut-off of 0.05 and log2 fold change more than 1, our analysis revealed 6361 differentially expressed genes (DEGs). 3506 genes were upregulated, and 2855 genes were downregulated DEGs (Supplementary Table S1 and Fig. 1). Non-coding RNA transcripts are about 90% of the eukaryotic genome and do not follow the central dogma for the flow of genetic information in cells. Although several studies aimed to analyse their existence, a significant challenge exists in terms of their molecular functions and mechanisms of action^42^. One of the rapidly expanding fields of this class of transcripts is the long non-coding RNA (lncRNA). A considerable number of transcripts (732) of lncRNA has been detected in our analysis, and interestingly, 513 of these transcripts had the highest (LFC is < − 3 or > 3) change in expression (Supplementary Table S2). None of the studied genes were included in our analysis due to the lack of a chicken-based biological pathway database for gene expression analysis.

**Table 1:** Summary statistics of RNA-Seq output.

Sample	Raw count	Cleaned count	Mapping %
Control 1	291,052,64	268,792,54	88.34
Control 2	113,657,819	102,102,063	87.89
Control 3	413,589,26	387,238,58	88.54
Challenged 1	915,321,41	872,570,38	90.42
Challenged 2	145,682,503	137,041,556	88.67
Challenged 3	140,667,468	125,491,032	90.23

The mapping percentage was calculated as the number of reads mapped to the reference genome divided by the number of cleaned count reads.

**Figure 2:**
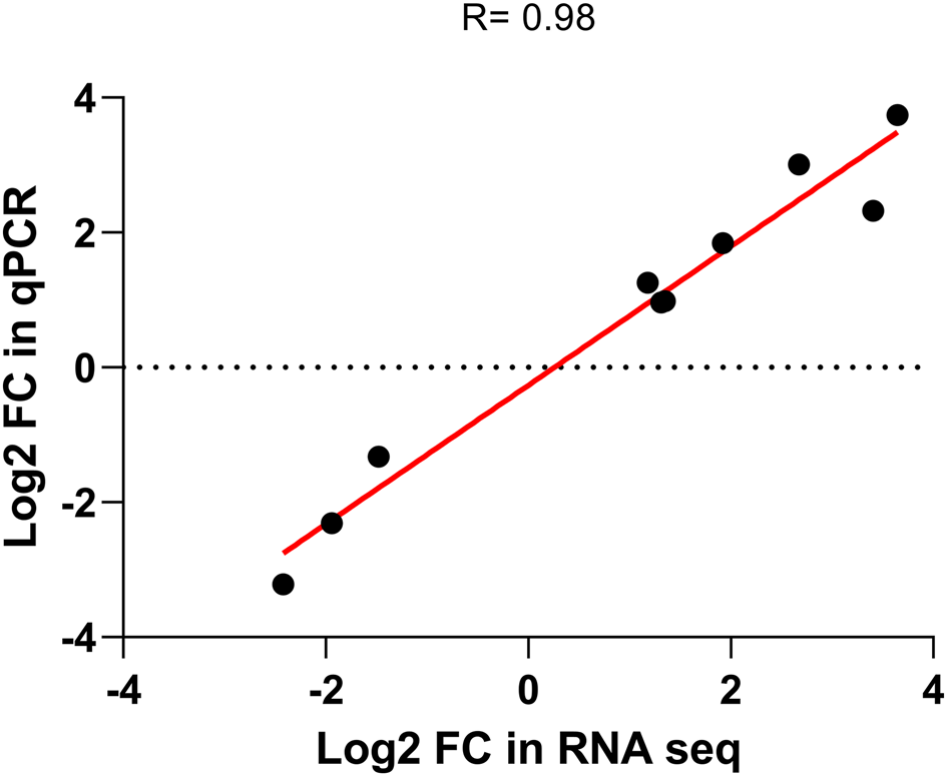
Validation of RNA-Seq data using ABI Quant studio qPCR system. The mean expression of ten selected genes was calculated by − ΔΔCT method and normalised by mean of *Ct* values of *YWHAZ* and *TBP YWHAZ* as reference genes. The values were converted into log_2_ fold change (LFC). Each dot point represents one gene. Pearson correlation coefficient test used to compare the results and its value labelled as “R”. Plus (+) and minus (−) signs indicate log_2_ FC values for the upregulated and downregulated genes, respectively.

Based on functional importance genes known to be involved in cell death^23,43^, ten genes were selected from our DEGs list to validate the RNA-Seq data. The selected genes were covered the full range of log_2_ fold change (log_2_ FC) and measured their expression level in qPCRs. The log_2_ FC obtained from RNA-Seq data analysis was compared to the log_2_ FC obtained in qPCRs. Figure 2 shows a comparison between the result of qPCR and RNA-Seq data. The expression patterns obtained from qPCR results for all ten selected genes were similar to their patterns from RNA-Seq analysis, with a correlation coefficient (R) of 0.98. These results confirming the reliability of the RNA-Seq data for gene expression patterns. Functional analysis of 6361 DEGs detected in NDV challenged chickens with IPA indicated the roles in immune response (specifically in early stage of splenic response) to the infection for most of top DEGs with z-score more than 3 (Table 2). Due to the use of human- and mouse-based database by IPA for analysis, the types and the functions of some of chicken genes have not been indicated properly. Further investigation into the functions of these uncharacterised proteins and genes would be useful to provide more insight into their contribution to infection. A list of DEGs with consistent responses in expression to NDV infection was released by Zhang et al.^21^. Comparison of DEGs in our study with Zhang et al. revealed 23 shared genes (Table 3). Thirteen (56%) of these shared genes had consistent expression change in our study and study of spleen of Hy-Line Brown birds^21^, spleen^20,44,45^, Harderian gland^16,19^, lung^17^, Trachea^18^ or embryo^46^ of Fayoumi or Leghorn chickens challenged with lentogenic NDV. Particularly, interferon induced protein with tetratricopeptide repeats 5 (*IFIT5*) showed significant up-regulation in the spleen of all chickens challenged with virulent and non-virulent NDVs. However, 10 (44%) of these shared significant DEGs had opposite regulation in our study, suggesting a quite different response to virulent NDV infections.

**Table 2:** The list of the genes that significantly (z-score > 3) affected at the challenged group.

Symbol	Function of gene^a^	LFC^b^	FDR^c^	Type(s)	HGNC^d^
*AGT*	Angiotensinogen	14.366	0.0009	Growth factor	183
*CAMK2A*	Calcium/calmodulin dependent protein kinase II alpha	10.169	0.0008	Kinase	815
*CAMKV*	CaM kinase like vesicle associated	10.958	0.0101	Kinase	79,012
*ELOVL2*	ELOVL fatty acid elongase 2	10.402	0.0019	Enzyme	54,898
*GABRA3*	Gamma-aminobutyric acid type A receptor subunit alpha3	10.432	0.0006	Ion channel	2556
*GFAP*	Glial fibrillary acidic protein	12.885	0.0004	Other	2670
*GPM6A*	Glycoprotein M6A	12.11	0.0020	Ion channel	2823
*IRX1*	Iroquois homeobox 1	10.723	0.0026	Transcription regulator	79,192
*MMD2*	Monocyte to macrophage differentiation associated 2	11.067	0.0016	Kinase	221,938
*PACSIN1*	Protein kinase C and casein kinase substrate in neurons 1	10.774	0.0009	Kinase	29,993
*PADI3*	Peptidyl arginine deiminase 3	10.267	0.0005	Enzyme	51,702
*PLP1*	Proteolipid protein 1	13.157	0.0003	Other	5354
*SLC15A2*	Solute carrier family 15 member 2	10.478	0.0022	Transporter	6565
*SLC1A3*	Solute carrier family 1 member 3	13.277	0.0011	Transporter	6507
*SLC6A11*	Solute carrier family 6 member 11	11.937	0.0014	Transporter	6538
*TTLL2*	Tubulin tyrosine ligase like 2	10.69	0.0016	Other	83,887

^a^IPA software was used to obtain gene’s function from the transcript identifier.

^b^LFC, Log twofold change.

^c^FDR, false discovery rate.

^d^HGNC, Human Gene Nomenclature Committee.

**Table 3:** Comparison of DEGs response to NDV in the present study and other in vivo NDV infection studies. LFC stands for log_2_ fold change.

Gene name	Function	LFC in this study at 2 dpi	Comparison with response in other NDV studies
*PLCXD1*	Phosphatidylinositol specific phospholipase C X domain containing 1	0.47	Consistent with spleen of Hy-line brown at 2 dpi^21^ and Harderian gland of leghorn at 6 dpi^19^
*SLBP*	Stem-loop binding protein	0.85	Consistent with spleen of Hy-line brown at 2 dpi^21^ and spleen^20^ and trachea^18^ of leghorn at 2 dpi
*OSTM1*	Osteoclastogenesis associated transmembrane protein 1	1.00	Consistent with spleen of Hy-line brown at 2 dpi^21^ and trachea of leghorn at 2 dpi^18^
*DRAM1*	DNA damage regulated autophagy modulator 1	1.40	Consistent with spleen of Hy-line brown at 2 dpi^21^ and Trachea of fayoumi and leghorn at 2 dpi^18^
*PARP12*	Poly(ADP-ribose) polymerase family member 12	1.48	Consistent with spleen of Hy-line brown at 2 dpi^21^ and harderian gland at 2 and 6 dpi^19^, in spleen at 2 dpi^20^ in leghorn
*SNX10*	Sorting nexin 10	1.90	Consistent with spleen of Hy-line brown at 2 dpi^21^ and spleen^20^ and trachea^18^ of leghorn at 2 dpi
*IFIT5*	Interferon induced protein with tetratricopeptide repeats 5	6.09	Consistent with spleen of Hy-line brown at 2 dpi^21^ and spleen of leghorn at 1^44^, 2^20,44^and 6 dpi^20^, and of fayoumi at 2 dpi^20^
*P2RX1*	Purinergic receptor P2X 1	− 5.62	Consistent with spleen of Hy-line brown at 2 dpi^21^ and lung of fayoumi at 10 dpi^17^
*KAZALD1*	Kazal type serine peptidase inhibitor domain 1	− 3.46	Consistent with spleen of Hy-line brown at 2 dpi^21^ and trachea of leghorn at 6 dpi^18^
*HPSE2*	Heparanase 2 (inactive)	− 2.61	Consistent with spleen of Hy-line brown at 2 dpi^21^ and trachea of leghorn at 2 dpi^18^
*UROC1*	Urocanate hydratase 1	− 2.52	Consistent with spleen of Hy-line brown at 2 dpi^21^ and harderian gland of leghorn at 6 dpi^19^
*ROR1*	Receptor tyrosine kinase like orphan receptor 1	− 1.66	Consistent with spleen of Hy-line brown at 2 dpi^21^ and lung of fayoumi at 2 dpi^17^
*FSHR*	Follicle stimulating hormone receptor	− 0.46	Consistent with spleen of Hy-line brown at 2 dpi^21^ and trachea of leghorn at 6 dpi^18^
*AICDA*	Activation induced cytidine deaminase	− 8.98	Inconsistent with spleen of Hy-line brown at 2 dpi^21^ and trachea of leghorn at 6 dpi^18^
*P2RY8*	P2Y receptor family member 8	− 3.24	Inconsistent with spleen of Hy-line brown at 2 dpi^21^ and harderian gland at 6 dpi^19^ and trachea at 2 and 6 dpi^18^ in leghorn
*ARHGAP15*	Rho GTPase activating protein 15	− 1.85	Inconsistent with spleen of Hy-line brown at 2 dpi^21^ and trachea of fayoumi at 2 dpi and leghorn at 2 and 6 dpi^18^
*ASNS*	Asparagine synthetase (glutamine-hydrolyzing)	− 0.58	Inconsistent with spleen of Hy-line brown at 2 dpi^21^ and harderian gland of leghorn at 6 dpi^19^
*TRIM24*	Tripartite motif containing 24	− 0.57	Inconsistent with spleen of Hy-line brown at 2 dpi^21^ and trachea of leghorn at 6 dpi^18^
*CDC42SE2*	CDC42 small effector 2	− 0.37	Inconsistent with spleen of Hy-line brown at 2 dpi^21^ and trachea of fayoumi at 2 dpi^18^
*BFAR*	Bifunctional apoptosis regulator	− 0.36	Inconsistent with spleen of Hy-line brown at 2 dpi^21^ and trachea of fayoumi and leghorn at 2 dpi^18^
*ST3GAL4*	ST3 beta-galactoside alpha-2,3-sialyltransferase 4	0.28	Inconsistent with spleen of Hy-line brown at 2 dpi^21^ and harderian gland of leghorn at 6 dpi under heat stress^16^
*MYH10*	Myosin heavy chain 10	0.29	Inconsistent with spleen of Hy-line brown at 2 dpi^21^ and trachea of leghorn at 6 dpi^18^
*EPHB1*	EPH receptor B1	0.32	Inconsistent with spleen of Hy-line brown^21^ and trachea of fayoumi at 2 dpi^18^

### Ingenuity pathway analysis of differentially expressed genes

**Figure 3:**
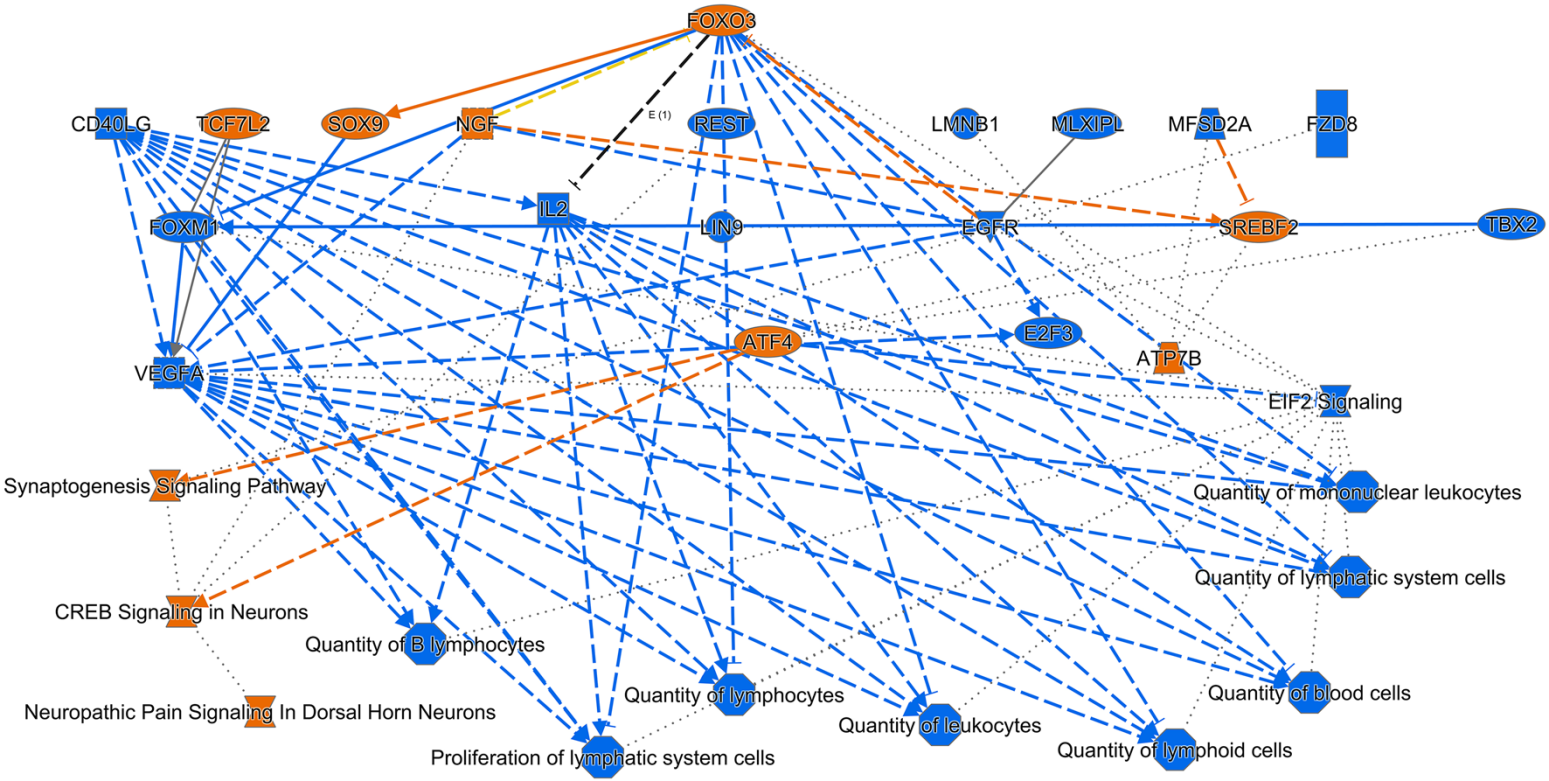
Major biological themes (pathways, upstream regulators, disease and biological functions) obtained from mapping the significantly upregulated DEGs in the spleen of chicks post infection. In IPA, only significantly enriched entities that passed a Fisher’s exact test p-value cut of 0.05 and also passes an absolute z-score cut-off of 2 or greater were visualised. Orange nodes are predicted to be activated (z-score ≥ 2), while blue nodes are predicted to be inhibited (z-score ≥ 2). Blue line: leads to inhibition, orange line: leads to activation green lines: decreased measurement.

**Figure 4:**
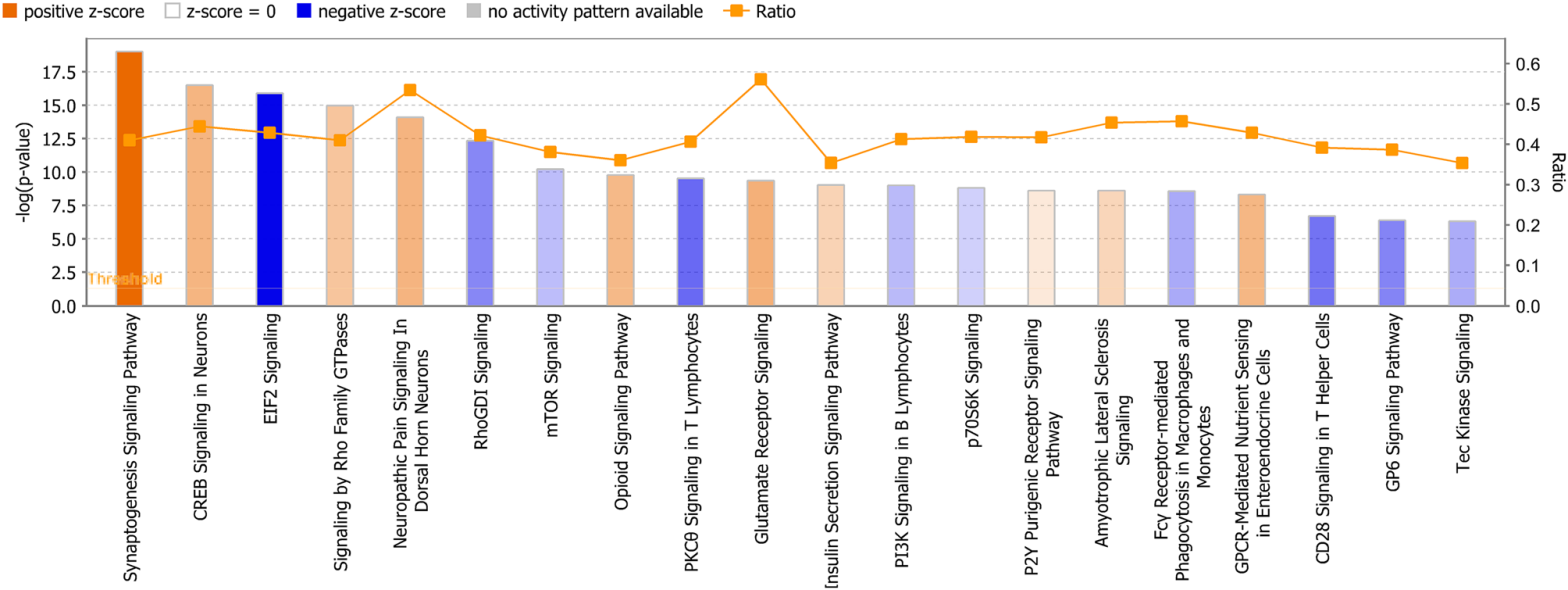
Top pathways of differentially expressed genes (FDR < 0.05). Pathways [Z-score > 0.05, − log (*p* value) > 1.3] in orange predicted to be activated and pathways in blue predicted to be inhibited. The more intensity of the colours, the higher absolute z-score. The proportion of genes within the pathways that were differentially expressed are indicated by the orange line as ratios. The height of each bar refers to the − log (p-value).

The DEGs list created from transcriptome analysis (p < 0.05, log2 fold change ≥ 1) was used as input for IPA analysis. IPA uses a Fisher’s exact test p-value cut of 0.05 and an absolute z-score cut-off of 2 or greater for pathways to consider them as significantly enriched. In this study, we focused on the pathways engaged in cell death and injury pathways. Overall canonical pathways, upstream regulators, disease and biological functions that were predicted by IPA to be activated are shown in Fig. 3. Inhibition of IL2 and downregulation of EIF2 signalling as an upstream regulator, resulted in the inhibition of B lymphocytes, the number of cells of the lymphoid system, mononuclear leukocytes and proliferation of cells of the lymphoid system. Inhibition of these processes may result in depletion in immune cells and lymphatic tissue destruction in spleen observed in spleen in challenged chicken with virulent NDV-GVII. IPA predicted upregulation of ATF4 that resulted in activation of synaptogenesis signalling pathway, CREB signalling in neurons and neuropathic pain signalling in dorsal horn neurons in our analysis. Several pathways were significantly impacted by the challenge with virulent NDV as predicted by IPA. Top pathways are shown in Fig. 4 and a list of all altered pathways provided in Supplementary Table S3. Overall, many of these pathways lead to cell death and immune response to infection. In particular, Elf2 and mTOR signalling was on top of our downregulated pathways. mTOR signalling activates autophagy and increased autophagy assistances NDV replication^47^. ElF2 pathway results in inhibition of viral replication through inhibition of translation of viral proteins and increased apoptosis in infected cells^48^. Reduced autophagy and increased apoptosis would help infected cells with the virus. IPA also predicted activation of signalling by Rho family GTPases, CREB signalling in Neurons and synaptogenesis pathway.

Zhang et al. study has recently reported 31 pathways with consistent expression response to non-virulent strains of NDV^21^. Comparison of predicted pathways in our study with impacted pathways by lentogenic NDVs, revealed 16 shared pathways (Table 4). Significant downregulation (z score < − 2) of 5 (31%) of the shared pathways in our study was in agreement with a study of NDV infection caused by a lentogenic strain in spleens of Hy-Line Brown chickens^21^. However, significant activation of these pathways has been reported in lung^17^, Harderian gland^19^ and trachea^18^ of Fayoumi and Leghorn chicken challenged with lentogenic strain of NDV. Eleven (68%) of these shared pathways also have not been significantly impacted (z score > − 2 or < 2) by virulent NDV in our study, while Zhang et al.^21^ and Deist et al.^17,18^ studies reported significant activation and inhibition of these pathways respectively in the response of lentogenic NDV. These findings suggest a different response of the immune system to virulent and non-virulent NDV in different tissues. Analysis of disease biomarkers in our results revealed functions associated with virulent NDV (Table 5). A decrease in proliferation of cells of the lymphoid system, quantity of B lymphocytes and quantity of mononuclear leukocytes are predicted by IPA using Ingenuity Knowledge Base approach^49^. Inhibition of these pathways together may contribute to the massive depletion of lymphoid cells in spleen observed in experimentally infected birds. However, an increase in microtubule dynamics is also predicted to be associated with NDV infection. Upregulation of key genes such as angiotensinogen (*AGT*) and proteolipid protein 1 (*PLP1*) contributed to both activated and inhibited pathways.

**Table 4:** Comparison of predicted pathways by IPA in current study with other studies that investigated the response to NDV infection.

zPathways	z-Score (current study)	IPA prediction in other studies using non-virulent NDV	
		Inhibition	Activation
TNFR2 signalling	− 3.00	Spleen^21^	Trachea^18^
TNFR1 signalling	− 2.32	Spleen^21^	Trachea^18^
GP6 signalling pathway	− 2.23	Spleen^21^	Harderian gland^19^
Leukocyte extravasation signalling	− 2.21	Spleen^21^	Trachea^18^
Production of nitric oxide and reactive oxygen species in macrophages	− 2.02	Spleen^21^	Trachea^18^
Fcγ receptor-mediated phagocytosis in macrophages and monocytes	− 1.54	Spleen^21^	Trachea^18^
Tec kinase signalling	− 1.50	Spleen^21^	Lung^17^, trachea^18^
B cell receptor signalling	− 1.13	Spleen^21^	trachea^18^
Integrin signalling	− 0.92	Spleen^21^	Lung^17^
IL-8 signalling	− 0.53	Spleen^21^	Lung^17^, trachea^18^
CD40 signalling	− 0.42	Spleen^21^	Trachea^18^
Thrombin signalling	− 0.12	Spleen^21^	Lung^17^
IL-6 signalling	0.18	Spleen^21^	Trachea^18^
P2γ purigenic receptor signalling pathway	0.88	Spleen^21^	Lung^17^
Relaxin signalling	1.00	Spleen^21^	Lung^17^
Ephrin receptor signalling	1.76	Spleen^21^	Lung^17^

Minus z-score means inhibition and positive z-score means activation.

**Table 5:** Top disease and biological function predicted by IPA to be associated with NDV infection.

Disease and functions	Contributed DEGs for prediction	z-Score	No.
Proliferation of cells of the lymphoid system	***PLP1****, ****SOX2, GAD2, UNC119, APOH, GAD1, MBP, ADCYAP1,**** FOXJ1, ****TYR***	− 3.571	168
Quantity of B lymphocytes	*SLCO1A2, ****F3****, ST6GALNAC2, ****FZD9****, HGF, ****PLCD1, ESR1****, BST1, ****ABL1****, ****STAT1***	− 2.769	93
Quantity of lymphocytes tissue	***GAD2****, NPY, SLC4A4, FADS2, FOXC2, ****PCSK1****, YES1, ESR1* ***ABL1****, MXI1*	− 2.402	89
Quantity of mononuclear leukocytes	***AGT, PLP1, GAD2****, ****MBP****, ADCYAP1, SLCO1A2, ****F3*** ***SNCA****, NPY, ****SLC4A4***	− 2.785	172
Microtubule dynamics	***AGT, GPM6A, PACSIN1, CAMK2A****, SOX2, PHGDH, RFX4, SLC39A12, ****GRIN1****, SNCB*	4.311	245

Bold italic and italic text indicates upregulated and downregulated DEGs respectively. Genes are sorted ascendingly from left to right based on their fold change. No. means the number of DEGs in our data contributed to disease production.

## Discussion

Understanding the molecular basis of pathogenesis of newly emerged NDVs will provide more reliable information on how these viruses produce unique pathological features in infected chickens, even in vaccinated flocks^8^. These information will provide more clear view on further vaccine development for protection of Newcastle disease worldwide. Gene expression pattern analysis helps to understand the virus and host interactions. Several in vivo and in vitro studies investigated gene expression in different tissues from experimentally infected chickens with different genotypes of NDV^17–22^. However, the molecular pathogenesis of genotype VII of NDVs has not been well described especially in in vivo studies. Herein, RNA-Seq and bioinformatics analyses were employed to study spleen transcriptome in experimentally infected birds with highly virulent NDV-GVII.

The viral strains used in this study were even more virulent than anticipated. The SPF chickens were inoculated via intranasal or intraocular rout with 10^2^ EID_50_ of GVII NDV and mortality began within 1 day after virus inoculation. The study had to be terminated on the second day, due to severe sickness of the birds and mortality.

In comparison, Alexander et al.^50^ inoculated poults with 10^6^ EID_50_ viscerotropic velogenic NDV-GIV (a 10,000 fold greater comparative dose), and clinical signs did not begin until the second day, with some birds surviving for 4 days^50^.

The spleen plays a critical role in early stage of the host defence response to NDV. Gene expression profile analysis of spleen provides insights into host immune defence. Splenic cells produce alpha and beta interferon and interleukin 6 (IL‐6) within the first 6 h of exposure of chickens to virulent NDV^51^. Spleen also has an important role in T cell immune response and lymphocyte transformation in immune response to NDV infection^52^.

In this study, a higher number of differentially expressed genes (> 6000) were found when compared to in vivo studies of nonvirulent NDV^17,18^. Liu et al. reported 8433 DEGs in chick embryo fibroblasts (CEFs) infected with virulent Herts/33 strain^22^. Regardless of the fundamental differences with our study, both studies showed similar patterns of gene expression with high number of DEGs in the response to virulent NDVs.

IPA predicted inhibition of mTOR and EIF2 signalling and placed them on top 10 pathways altered by NDV infection in our list. mTOR signalling regulates CD8 T cell differentiation^53^, and induces Toll-like receptor-mediated IFNA1 in plasmacytoid dendritic cells and has a negative control role in autophagy-mediated cell death after viral infection^54,55^. mTOR signalling activates autophagy and an increased autophagy benefits NDV replication^47^. EIF2 signalling has been known as a viral replication inhibitor and proinflammatory cytokine expression regulator^56^. EIF2 pathways inhibits translation of virus and increases apoptosis in infected cells, resulting in inhibition of viral replication^48^. Downregulation of these pathways indicates the host’s immune response in preventing of viral replication in infected cells. Deist et al. reported downregulation of EIF2 pathway in lung of challenged Fayoumis with lentogenic NDV at 10 dpi^17^. However, different pattern of activation of EIF2 signalling pathway were reported in trachea and spleen of challenged birds with non-virulent NDV at 2 dpi^18^, and viral shedding were not reported in these studies. Considering the crucial role of these pathways in inhibition of viral replication, downregulation of viral replication pathways may indicate that the host used this strategy to defend itself from the virulent NDVs.

Our IPA analysis also indicated the downregulation of some shared immune pathways with other in vivo NDV infections^21^. IL-8 signalling has a vital role during infectious disease by regulation of chemotaxis and activation of neutrophils^57^. IL-15 production also facilitates homeostasis, development of natural killer cells and CD8 T cells during anti-viral response^58^. Tec kinase signalling pathway has also critical role in response to viral infection and is essential for differentiation and development of CD4+^59^ and CD8+ T cell^60^. IL-2 has a critical role in activation of NK cells, lymphocyte proliferation and clearance of intracellular pathogens in chickens^61,62^. Inhibition of these share pathways and especially IL-2, as a key upstream regulator in our study, suggests a suppressed immune response caused by this newly emerged NDV-GVII.

Most of the top upregulated genes indicated in our RNA transcriptome were involved in the immune response to the infection in spleen. AGT and PLP1 both are associated with increased quantity of cytotoxic CD8+ T-cell^63^. GPM6A has a role in the expression of human GPM6A mRNA in marginal-zone B lymphocytes expressing human CD27 protein and human IgD complex^64^. Upregulation of AGT and PLP1 in our study contributed in disease production that resulted in massive depletion of spleen.

Previous studies reported tissue-specific immune response^65^ and breed specific immune gene expression in chickens^66^. Our results indicate small portion and also opposite regulation of shared significant DEGs with previous in vivo studies of lentogenic NDV. These differences may be due to infection caused by a virulent NDV in our study and emphasise the host's different immune response to the virulent and non-virulent viruses.

Our result shows activation of pathways that regulate cellular actin such as signalling by Rho family GTPases resulting an activated microtubule dynamics pathway. The critical role of this pathway in cell–cell fusion and syncytium formation in pathogenesis of paramyxoviruses that helps virus entry to the host cell has been reported by Gower et al.^67^. These results may suggest that NDV as a paramyxovirus, facilitates viral replication and infection by activation of this pathway.

IPA analysis also pointed out activation of synaptogenesis signalling in our results. This pathway plays a critical role in development of nervous system by regulation of synapse formation between neurons^68^.

Gastrointestinal lesions and severe atrophy of the lymphoid organs due to necrosis and depletion of the lymphocytes has been reported in the infection caused by the velogenic viscerotropic NDVs^69,70^. Severe lymphocytic destruction in the lymphoid organs can also lead to immunosuppression in survived chicken from NDV infection^71^. Severe atrophy of the spleen has been described in other immunosuppressive disease of chickens such as infectious bursal disease^72,73^. Our IPA analysis indicate a decreased quantity of lymphoid tissue and inhibited proliferation of cells of the lymphoid system, which was in agreement with our gross pathology observation of lymphocyte depletion in spleen. These evidences may supports possible immunosuppressive effects of this strain.

Activation of pathways that results in development of synapses in nervous system and depletion of lymphoid tissue suggests possible the host response to this strain. More in situ detection and analysis of viral antigens in different tissues of infected birds is necessary for comprehensively understanding of the tissue tropism of this newly emerged virulent NDV.

On the other hand, our results indicate genes with consistent expression regulation in different studies with many varied experimental factors such as virulence of virus, tissue and breed and time point sampling. This suggests a universal role of these genes in immune response to NDV. One of the most significant genes is IFIT5, which is an interferon-stimulated gene and its critical role for innate immune defence against virus has been confirmed^74^. IFIT5 recognises and inhibits translation of viral RNA bearing a 5′-triphosphate^75^, and also has a key regulator role in activation of B-cells by positive regulation of nuclear factor kappa-light-chain-enhancer in NF-кB signalling pathway^76^. Overexpression of IFIT5 in transgenic chickens showed significant enhanced resistance to avian influenza and velogenic NDV^77^. Consistent up-regulation of IFIT5 in the spleen of all chickens challenged with virulent and non-virulent NDV indicates critical role of this gene in splenic immune response to viral infections.

Compared to the great importance of NDV to the poultry industry and its effects on international trade, there is a relatively modest number of published infectious challenge experiments in which virulent virus has been administered to chickens. In vivo experiments using virulent NDV require specialised animal PC3 facilities which are expensive to build and operate. Rapid progression of induced disease can make it difficult to sample birds over multiple days post infection. In the present study, we had intended to sample birds at 48 and 72 h post-inoculation. However, we decided to humanely kill them all at 48 h post challenge due to severe clinical signs of disease and high mortality that were already increasing within 24 h. The reduced quality of RNA extracted from infected birds in comparison with healthy birds was another limitation of this study that was an unavoidable consequence of destruction of the host transcriptome during the acute phase of paramyxovirus infection^78^.

## Conclusion

This is the first study of gene expression profiling of spleen tissue of experimentally infected chickens with a virulent NDV-GVII. In conclusion, we observed extensive alteration of gene expression in response to this strain in the spleen of chickens. Multiple comparisons of gene expression profile of spleen between this study and previous studies of lentogenic NDV infections indicates differences between DEGs and activation pathway patterns, indicating the role of virulence of virus in immune responses. Activation of autophagy-mediated cell death, lymphotropic and synaptogenesis development pathways after viral infection suggests a new tissue tropism for genotype VII NDVs. Further in vivo study of these virulent NDV strains in chickens is needed to more comprehensively reveal the molecular pathogenesis of these newly emerged virulent strains of NDV.

## Materials and methods

### Viruses

The challenge strain used in this study (Mega) has previously been characterised by measuring the MDT index according to standard OIE manual procedures^10^. The mean death time (MDT) of the isolate was 30 h that classified the isolate as virulent or velogenic viruses^11^. In brief, ten-fold serial dilution between 10^−6^ and 10^−9^ of the virus was made in sterile PBS. A hundred microliters of each dilution were inoculated into the allantoic cavity of each of five 9-day-old embryonated SPF chicken eggs and incubate at 37 °C, monitored twice a day for 7 days. The time for any embryo deaths was recorded, and the minimum lethal dose that caused death in all embryos was calculated. The minimum lethal dose (MDT) is the highest dilution of the virus that causes death in all the embryos^79^. In our case, the MDT for the Mega strain of NDV was 30 h.

### Challenge experiment

Animal experiments were performed at the Indonesian research centre for veterinary science (Bbalitvet), Bogor, Indonesia. The animal ethics was approved by the research and animal ethics committee of Bbalitvet institute with reference number of AH/2015/003. An experienced veterinarian managed the challenge experiment in accordance with the National Health and Medical Research Council (NHMRC) of Australia and the Animal Research Reporting of In Vivo Experiments (ARRIVE) guidelines 2.0. Twenty, 1-day old SPF broiler Ross chickens sourced from Caprifarmindo Laboratories (Bandung, Indonesia) were divided into two groups of 10 and raised in isolator units at biosafety level 3 (BLS3) biocontainment at Bbalitvet. Chickens were allocated randomly into two isolators and tagged individually. At 35 days of age, the birds were inoculated by intraocular and intranasal instillation with100µL of 100 EID50^50,80^ of live Mega strain of NDV (accession number of MN688613)^11^. One group of 10 birds was kept as a negative control in isolator 2.

### Tissue collection and RNA extraction

Following viral challenge, birds were monitored twice a day for clinical signs of ND. Dead birds were collected and sampled immediately, and those with severe clinical signs of disease were euthanised and counted as mortalities for that day. Due to death or severe sickness of chickens in the challenged group, the experiment was terminated at 2 day post infection (2 dpi), and the birds in all groups were euthanised by cervical dislocation. After opening of the carcass, all tissue sample were collected from freshly necropsied chicken and immediately divided into four pieces for four different experiments (histopathology, virus isolation, RNA isolation and backup). The tissues for RNA extracting were immediately frozen in sterile tubes on dry ice and transferred either to RNA later or to Trizol reagent (Ambion, Thermo Fisher, MA, USA). In total, twenty RNA samples from spleens of challenged and control groups were isolated using a mirVana miRNA isolation kit (Ambion, Thermo Fisher, Lithuania). To remove the genomic DNA residue, the isolated RNA was treated with DNase using a DNA-free kit (Thermo Fisher Scientific, Carlsbad, CA, USA). RNA quality was assessed by Agilent 2200 TapeStation instrument, (Agilent Technologies, Santa Clara, CA, USA) and confirmed as RNA Integrity Number (RIN) > 5 for all samples.

### Detection of virus shedding in challenged and control chickens

A cloacal swab sample was taken from each chicken, and viral RNA extracted from cloacal swab samples using QIAamp Viral RNA Mini kit (Qiagen, Louisville, KY, USA) and quantified using NanoDrop 1000 Spectrophotometer (Thermo Fisher Scientific, Carlsbad, CA, USA). Five microliters of extracted RNA was converted to cDNA using a QuantiTect Reverse Transcription Kit (Qiagen, Louisville, KY, USA) as per manufacturer’s instruction. For detection of viral load in samples, an absolute quantification method used in qRT-PCR. NDV-Fusion-Forward: AAAGTGGTGACACAGGTCGG, and NDV-Fusion-Reverse primer: CCGATGTATTGCCGCTCAAG used for amplification of a 145 bp amplicon. A QuantiFast SYBR^®^ Green PCR Kit (Qiagen, Louisville, KY, USA) was used in a real-time polymerase chain reaction (RT-PCR) in an Illumina, Eco Real-Time PCR machine (California USA). The reaction was run in triplicate for each qPCR with an initial denaturation at 95 °C for 3 min followed by 45 cycles of 95 °C for 10 s and 60 °C for 30 s. The Ct values greater than 35 in viral samples were considered negative^81^. One-way analysis of variance (ANOVA) was undertaken to test for mean differences in CT values. The results were analysed in IBM SPSS (v 26.0; SPSS Inc., Chicago, IL).

### RNA sequencing

After the initial assessments and the quality control of the RNA samples, three RNA samples from the challenged group and three RNA samples from the negative control group with RIN > 5 were selected for the further analysis. The selected samples were submitted to Australian Genome Research Facility for RNA sequencing. Sequencing libraries were prepared with the TruSeq RNA Library Predation kit as per the manufacturer’s protocol and sequenced on Illumina NovaSeq 6000 platform.

### Transcriptome analysis

Raw RNA-Seq paired end reads were checked for quality using FASTQC v0.11.4^82^ and trimmed with TrimGalore v0.4.2^83^ to a minimum length of 150 bp per read and Phred score of 10. Sequencing adapters were removed with AdapterRemoval v2.2.1^84^. Cleaned reads were mapped to the chicken reference genome (GRCg6a) using Hisat2 v2.1.0^85^. Mapped reads were sorted with SAMtools v1.8^86^. Then, sorted mapped reads were summarised using FeatureCounts^87^ at the gene level using Ensembl annotation version 97. The Voom-limma pipeline^88,89^ was used to analyse samples grouped by infection status using the gene-level read counts as input. Briefly, the pipeline involved the removal of lowly expressed genes, i.e. genes meeting the requirement of count per million (CPM) more than one in at least three samples. The counts were normalised by log-transforming the counts per million to standardise for differences in library size. Counts were also normalised using trimmed mean of M values (TMM) method^90^ to avoid bias from different coverage, and samples and individual observational level of each expressed genes were weighted using Voom^89^ to account for heterogeneity in their expression level. Moderated t-statistics tests were used to define differential expression levels between samples. Differentially expressed genes (DEGs) between groups with different infection status were tested and ranked based on the false discovery rate (FDR) less than 0.05.

### Pathway analysis of differentially expressed genes

Differentially expressed genes (DEGs) with FDR < 0.05 were analysed using the Ingenuity Pathway Analysis software (IPA, QIAGEN, Redwood City, CA, USA), and pathways or functions with z-score > 2 were considered to be activated or inhibited^49^.

### Validation of RNA-Seq data

Applied Biosystems Real-Time PCR System comparative Ct (ΔΔCt) assay was used to validate RNA-Seq results. Gene expression measured in all tissues samples in challenged and control groups (n = 20). For each sample, cDNA was prepared from 1 μg of RNA using the QuantiTect Reverse Transcription Kit (Qiagen, Melbourne, Australia) according to the manufacturer’s protocols. PowerTrack SYBR Green Master Mix (Thermo Fisher Scientific, Australia) was used to prepare PCR master mix in a 20 μL reaction volume as per the manufacturer’s protocol and 2 μL of the cDNA was added into each reaction well (in triplicate) using a robot (Ep Motion 5075 Robot system, Eppendorf AG, Hamburg, Germany). Thermocycling conditions in ABI Quant Studio 6 Flex thermal cycler (Thermo Fisher Scientific, Australia) were polymerase activation at 95 °C for 2 min, 40 cycles of denaturation at 95 °C for 15 s, annealing at 60 °C for 60 s. A melting curve step from a ramp of 50–99 °C was included to assess the specificity of amplification. Based on their log2 FC (LFC) in RNA-Seq analysis, we selected ten genes that cover the full range of LFC in the comparisons between treatments and control group, and the functional importance of each gene in cell death has also been considered^23,43^. These primers are listed in Table 6. The primers were designed by NCBI primer tool with amplicons around 100 bp and spanning multiple exons specified also applied to avoid amplification of genomic DNA. uMelt web-based tool used for prediction of DNA melting curves and denaturation profile of PCR products for assessment of specific amplification of primers^91^. Amplifications of a series of five, ten-fold dilution of cDNA used for determination of PCR amplification efficiencies and correlation coefficients (R)^92–95^. The geometric mean of Ct values of YWHAZ and TBP housekeeping genes, that are more stably expressed in the spleen of chicken challenged with pathogens^96,97^, were used for normalisation of the data. Data were analysed using the Quant Studio Real-Time PCR Analysis software. Replicates of the same sample showing a shifted peak in melting curves were removed. Gene expression was compared between control and treatment groups using the 2(− ΔΔCt) method, and Pearson Correlation Coefficient between LFC in qPCR assay and RNA-Seq data were calculated, using the GraphPad prism software version 8.4.2 (GraphPad Software, LLC, San Diego, CA, USA).

**Table 6:** Primer sequence used in qPCR for RNA-Seq data validation.

Gene symbol	Primer sequence (5*'*–3*'*)	Exon junction (bp)	Fragment size (bp)	Annealing °C	PCR efficiency (%)	Correlation coefficient (R)	Slop	NCBI accession	References
*APAF1*	F: GGTCAATTGCTGCCAGTTCA	2316/2317 (reverse primer)	94	60	129	0.9539	− 2.76	XM_416167.6	This study
	R: TCCTTCAAATCCCAAAGTTTGAT								
*CASP3*	F: GCAGACAGTGGACCAGATGA	90/91 (reverse primer)	94	60	166	0.9502	− 2.349	XM_015276122.2	This study
	R: AGGAGTAGTAGCCTGGAGCA								
*CYCS*	F: CGTGGGCGCATTTACTGACA	107/108 (forward primer)	81	60	130	0.9650	− 2.759	XM_015281453.2	This study
	R: CCGTATGGCACTGGGAACAT								
*CASP9*	F: CGGAACCTCAAAGCTCAGGAAA	667/668 (forward primer)	99	60	158	0.9524	− 2.425	XM_424580.6	This study
	R: ATGGGAGAGGATGACCACGA								
*PMAIP1*	F: GCCTGCAGAGCGGGAC	114/115 (forward primer)	89	60	130	0.9580	− 2.756	NM_001302097.1	This study
	R: GGTTCAGGACTTTCTGCTGC								
*TP53INP1*	F: ACACTGGCACAACTGGAGG	813/814 (forward primer)	72	60	157	0.9520	− 2.429	XM_015282925.2	This study
	R: GGTAGGAAGAGCTGCGACAA								
*TP53INP2*	F: ATCGAGCTTGGAGAAGAGCC	527/528 (forward primer)	96	60	181	0.9418	− 2.227	XM_015296284.2	This study
	R: GGTGACGTAGACGGACATGC								
*TP53BP2*	F: CTGTGCAAGGAACCTGGTGA	326/327 (reverse primer)	74	60	152	0.9469	− 2.483	XM_419394.6	This study
	R: TCGGCTATAGGCCGTTCTGA								
*CLTA*	F: CTAGCAACAGGGTGGCAGAT	615/616 (reverse primer)	79	60	156	0.9495	− 2.440	XM_015280418.2	This study
	R: GCTTCTTCAGCTGCCACATAAC								
*MLKL*	F: ATTTGAAGGCTGCCCTCTCC	1216/1217 (forward primer)	121	60	206	0.9524	− 2.055	XM_015279230.2	This study
	R: GAAGGCCCGACACTGATTGA								
*TBP*^a^	F: CCACGGTGAATCTTGGTTGC	534/535 (reverse primer)	88	60	156	0.9423	− 2.447	NM_205103.1	^97^
	R: GCAGCAAAACGCTTGGGATT								
*YWHAZ*^a^	F: TTGCTGCTGGAGATGACAAG	E2/E3 (forward primer)	61	60	120	0.9656	− 2.910	NM_001031343.1	^98^
	R: CTTCTTGATACGCCTGTTG								

For calculating amplification efficiency, a standard curve was generated using a tenfold dilution of cDNA. The standard curve was created by plotting the Cq values against the log of the starting quantity of template for each dilution.

^a^Used as reference genes for relative expression data analysis. Exon junction represent the spanning of exon on genes sequence.

## Supplementary Information

Supplementary Tables.

## Data Availability

The raw RNA-Seq data have been deposited in NCBI SRA database under the BioProject accession number PRJNA675698.
